# Activity of Imipenem, Meropenem, Cefepime, and Sulbactam in Combination with the β-Lactamase Inhibitor LN-1-255 against *Acinetobacter* spp.

**DOI:** 10.3390/antibiotics10020210

**Published:** 2021-02-20

**Authors:** Cristina Lasarte-Monterrubio, Juan C. Vázquez-Ucha, Maria Maneiro, Jorge Arca-Suárez, Isaac Alonso, Paula Guijarro-Sánchez, John D. Buynak, Germán Bou, Concepción González-Bello, Alejandro Beceiro

**Affiliations:** 1 Servicio de Microbiología, Instituto de Investigación Biomédica de A Coruña (INIBIC-CICA), Complejo Hospitalario Universitario A Coruña (CHUAC), As Xubias 84, 15006 A Coruña, Spain; crlasarm@gmail.com (C.L.-M.); juan.vazquez@udc.es (J.C.V.-U.); guijarro.sanchez.p@gmail.com (P.G.-S.); German.Bou.Arevalo@sergas.es (G.B.); 2 Centro Singular de Investigación en Química Biolóxica e Materiais Moleculares (CIQUS), Departamento de Química Orgánica, Universidade de Santiago de Compostela, Jenaro de la Fuente s/n, 15782 Santiago de Compostela, Spain; maneirorey@gmail.com (M.M.); concepcion.gonzalez.bello@usc.es (C.G.-B.); 3 Servicio de Microbiología, Hospital Provincial Pontevedra, Loureiro Crespo 2, 36002 Pontevedra, Spain; Isaac.Alonso.Garcia@sergas.es; 4 Department of Chemistry, Southern Methodist University, Dallas, TX 75275, USA; jbuynak@mail.smu.edu

**Keywords:** *Acinetobacter* spp., *Acinetobacter baumannii*, β-lactam antibiotic resistance, β-lactamase inhibitors, LN-1-255, imipenem, meropenem, cefepime, sulbactam, carbapenem-hydrolyzing class D β-lactamases (CHDLs)

## Abstract

Treatment of infections caused by *Acinetobacter* spp., particularly *A. baumannii*, is a major clinical problem due to its high rates of antibiotic resistance. New strategies must be developed; therefore, restoration of β-lactam efficacy through the use of β-lactamase inhibitors is paramount. Activities of the antibiotics imipenem, meropenem, cefepime, and sulbactam in combination with the penicillin-sulfone inhibitor LN-1-255 were tested by microdilution against 148 isolates of *Acinetobacter* spp. collected in 14 hospitals in Spain in 2020. Relevantly, the MIC_90_ (i.e., minimum concentration at which 90% of isolates were inhibited) of antibiotics in combination with LN-1-255 decreased 4- to 8-fold for all of the *Acinetobacter* isolates. Considering only the carbapenem-resistant *A. baumannii* isolates, which produce carbapenem-hydrolyzing class D β-lactamases, the addition of LN-1-255 decreased the resistance rates from 95.1% to 0% for imipenem, from 100% to 9.8% for meropenem, from 70.7% to 7.3% for cefepime, and sulbactam resistance rates from 9.8% to 0% and intermediate susceptibility rates from 53.7% to 2.4%. The inhibitor also decreased the minimum inhibitory concentrations (MICs) when tested against non-carbapenem-resistant *Acinetobacter* spp. isolates. In conclusion, combining LN-1-255 with imipenem, meropenem, cefepime, and sulbactam to target *A. baumannii*, and especially carbapenem-resistant isolates, represents an attractive option that should be developed for the treatment of infections caused by this pathogen.

## 1. Introduction 

*Acinetobacter* is a highly diverse genus comprising both human pathogens and environmental microorganisms. Regarding human pathogens, the *Acinetobacter calcoaceticus*-*Acinetobacter baumannii* (ACB) complex (*A. calcoaceticus*, *A. baumannii*, *A. nosocomialis*, and *A. pittii*) is the group of most concern in terms of clinical importance. *A. baumannii* is currently one of the most important nosocomial pathogens [1], known to be associated with life-threatening infections in immunocompromised hosts and in patients with severe underlying diseases. Treatment and eradication are increasingly challenging not only because of the intrinsically high resistance of this pathogen but also because of its natural propensity to develop multidrug resistance, including resistance to carbapenems, via the horizontal acquisition of broad-spectrum resistance mechanisms [2].

Carbapenems, like imipenem and meropenem, are the main therapeutic options available to treat serious infections caused by *A. baumannii*, partly due to the ability of these antibiotics to withstand modifications by the naturally produced β-lactamases of *A. baumannii* (OXA-51 and ADC-type cephalosporinases), as well as their good target-binding properties and favorable safety profile [3]. Cephalosporins such as cefepime, a zwitterionic cephalosporin with some degree of stability to hydrolysis mediated by ADC β-lactamases [4] and with enhanced bacterial cell penetration, may still remain useful. Likewise, sulbactam, a class A β-lactamase inhibitor with a high affinity for the *A. baumannii* penicillin-binding protein 2 (PBP2) [5] and intrinsically bactericidal against this pathogen, is another possible treatment option.

Treatment of infections caused by *A. baumannii* is particularly challenging because of the propensity of this species to develop antimicrobial resistance through horizontal acquisition and/or upregulation of intrinsic mechanisms, and very few therapeutic options are currently available. Thus, acquired carbapenem-hydrolyzing class D β-lactamases (CHDLs), such as OXA-23, OXA-24/40, OXA-58, OXA-143, or OXA-235, are of great concern as they represent the main threat to the use of carbapenems, the first-line antibiotics available for managing *A. baumannii* infections [6]. Reduced permeability of the outer membrane and active efflux are also involved in carbapenem resistance [7]. Resistance to cefepime in *A. baumannii* can be mediated by alteration of the outer membrane properties, the presence of horizontally acquired β-lactamases, and the production of extended-spectrum AmpC β-lactamases, such as ADC-56 [8,9,10]. Finally, sulbactam resistance in *A. baumannii* is probably multifactorial and has been related to the expression of *bla*_OXA-23_ [11], *bla*_TEM-1D_, increased production of ADC (IS*Aba1*-*ampC*) [12], and reduced expression of PBP2 [13]. 

**Figure 1:**
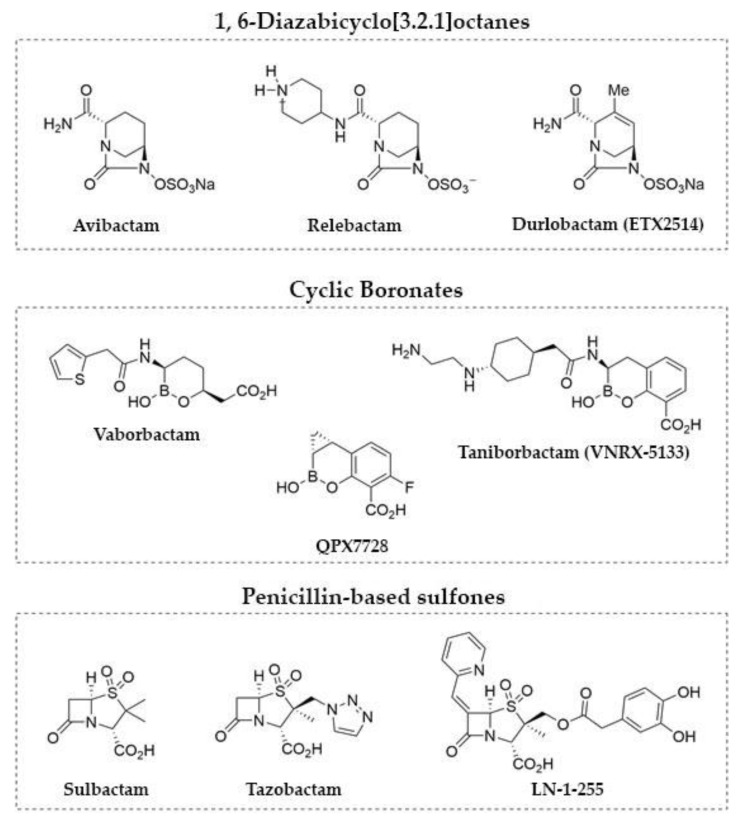
Selected examples of relevant β-lactamase inhibitors.

To date, β-lactamase inhibitors have been successfully used to restore the efficacy of β-lactam antibiotics for treatment of infections caused by β-lactamase-producing Gram-negative pathogens [14] (Figure 1). However, CHDLs produced by *A*. *baumannii* are recalcitrant to inhibition by classical (e.g., tazobactam, sulbactam, and clavulanate) or recent commercially available inhibitors (e.g., avibactam, relebactam, and vaborbactam) [15,16,17]. As a consequence, inhibition of CHDLs remains an unmet challenge regarding the use of β-lactams to treat severe *A. baumannii* infections. In this regard, the emergence of new broad-spectrum inhibitors, mainly durlobactam (formerly ETX2514), a 1,6-diazabicyclo[3.2.1]octane [18]; QPX7728, a cyclic boronate [19]; and LN-1-255, a penicillin sulfone derivative (all of which are able to block the most widespread CHDLs produced by *A*. *baumannii*) may represent a step forward in the fight against infections caused by β-lactam-resistant and, in particular, carbapenem-resistant *A. baumannii*.

LN-1-255 is a 6-alkylidene-2′-substituted penicillin sulfone inhibitor with demonstrated activity against class A, class C, and class D β-lactamases [20,21] and against the carbapenem-hydrolyzing oxacillinases produced by *A. baumannii.* This inhibitor presents a catechol moiety responsible for effective internalization via bacterial iron uptake pathways. LN-1-255 is anchored in the active site of β-lactamases by strong electrostatic and hydrogen-bonding interactions between the sulfinate group and the carbamoyl group of the inhibitor and diverse polar residues within the pocket of the enzymes [17]. 

In previous research, we observed that relative to tazobactam and avibactam, LN-1-255 wields significant in vitro inhibitory activity against isogenic *A. baumannii* strains carrying OXA-23, OXA-24/40, OXA-58, OXA-143, and OXA-235 CHDLs enzymes, displaying LN-1-255 approximately three logs higher affinity for CHDLs (*K_I_*) than comparators [22]. Murine pneumonia models were likewise used to test the in vivo performance of this penicillin sulfone inhibitor, with promising results being obtained in terms of toxicity and reduction of the bacterial burden relative to imipenem monotherapy in mice [23]. 

National and international surveillance studies are important for determining the in vitro activity of newly developed antimicrobials. These traditional approaches are useful to evaluate and control antimicrobial resistance trends and for guiding decisions regarding appropriate treatments. Nevertheless, LN-1-255 activity has not been tested with large collections of *Acinetobacter* spp. clinical isolates. Therefore, the aim of the present study was to evaluate whether LN-1-255 enhances (restores) the activity of imipenem, meropenem, cefepime, and sulbactam against a collection of *Acinetobacter* spp. clinical isolates recovered from 14 hospitals across Spain in 2020, in order to confirm the therapeutic potential of this β-lactamase inhibitor and to determine the best LN-1-255/antibiotic combination.

## 2. Results and Discussion

Antimicrobial susceptibility testing by reference broth microdilution was performed to determine the minimum inhibitory concentrations (MICs) for imipenem, meropenem, cefepime, and sulbactam alone or in combination with the inhibitor LN-1-255. Important differences in rates of resistance to the antibiotics tested were observed in relation to the *Acinetobacter* species and CHDLs production, as might be expected. Resistance rates to the four antibiotics were higher in *A. baumannii* strains carrying acquired CHDLs. For this reason, and for the purpose of simplicity, the results of this study are presented for the whole collection of isolates and separately for CHDL-producing *A. baumannii* isolates, non-CHDL-producing *A. baumannii*, and all of the non-*A. baumannii* isolates (which included isolates of *A. calcoaceticus*, *A. dispersus*, *A. nosocomialis*, *A. dijkshoorniae*, *A. ursingii*, *A. pittii*, *A. guillouiae*, *A. johnsonii*, and *A. bereziniae*).

### 2.1. Carbapenems/LN-1-255

Carbapenem resistance rates in the whole set of 148 clinical isolates of *Acinetobacter* spp. were 28.4% for imipenem and 35.1% for meropenem (following CLSI clinical breakpoints) and no important differences in MIC_50/90_ values were observed (MIC_50/90_ ≤ 0.5/16 and ≤0.5/32, respectively) (Table 1 and Table 2).

**Figure 2:**
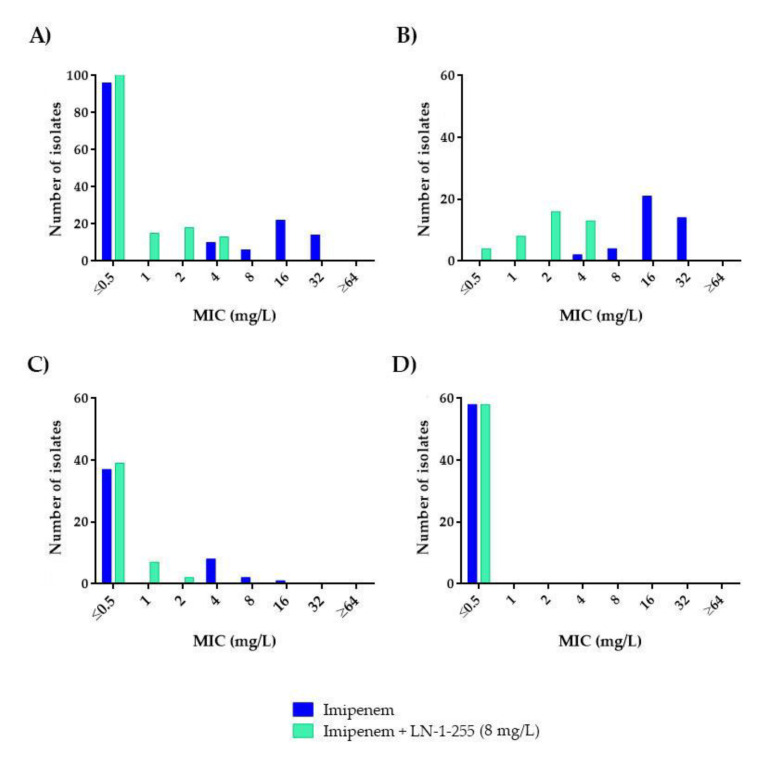
Comparative analysis of imipenem and imipenem/LN-1-255 MICs (mg/L): (**A**) All isolates (*n* = 148), (**B**) CHDL-producing *A. baumannii* (*n* = 41), (**C**) *A. baumannii* producing only OXA-51-like (*n* = 48), and (**D**) non-*A. baumannii* (*n* = 59).

**Figure 3:**
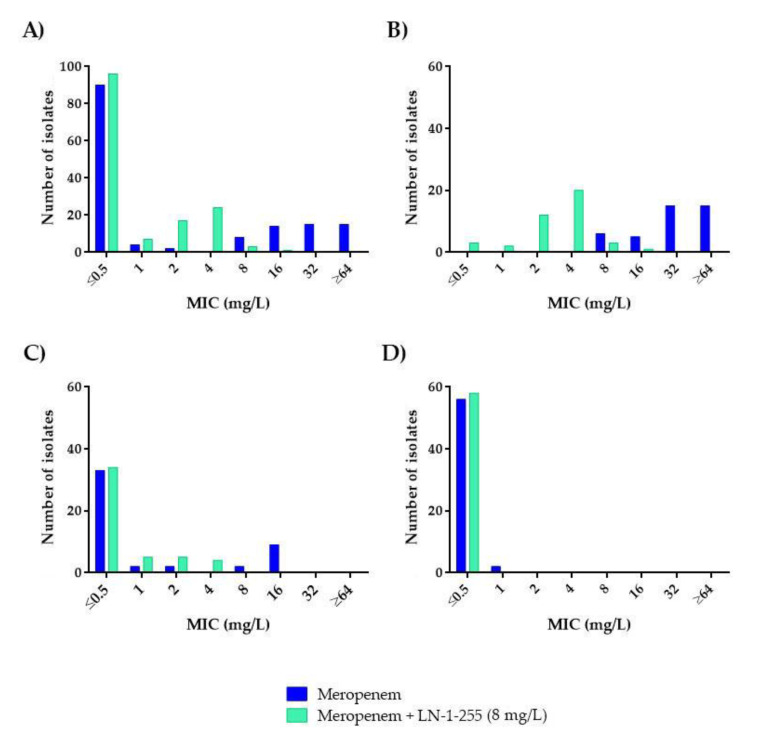
Comparative analysis of meropenem and meropenem/LN-1-255 MICs (mg/L): (**A**) All isolates (*n* = 148), (**B**) CHDL-producing *A. baumannii* (*n* = 41), (**C**) *A. baumannii* producing only OXA-51-like (*n* = 48), and (**D**) non-*A. baumannii* (*n* = 59).

Susceptibility to carbapenems was very different in the CHDL-producing *A. baumannii* subgroup (*n* = 41), which mainly harbored *bla_OXA-23-like_* (*n* = 34, 82.92%) (Table 3), than in the *A. baumannii* isolates lacking acquired CHDLs (*n* = 48) and the non-*A. baumannii* isolates (*n* = 59). None of the non-*A. baumannii* isolates were CHDL producers. None of the *A. baumannii* strains with acquired CHDLs were considered fully susceptible to imipenem or meropenem, to which 95.1% and 100% of the isolates were resistant, respectively. Among the non-CHDL-producing *A. baumannii* isolates, resistance rates decreased to 6.3% and 22.9% for imipenem and meropenem, respectively. No carbapenem-resistant isolates were detected in the non-*A. baumannii* species (Figure 2 and Figure 3). 

Addition of LN-1-255 at a fixed concentration of 8 mg/L decreased the carbapenem resistance rates in all the *A. baumannii* strains tested (Table 1 and Table 2). For the group of acquired CHDL-producing *A. baumannii*, imipenem and meropenem decreased the resistance rates from 95.1% to 0% and from 100% to 9.8%, respectively, decreasing the MIC_50_ and MIC_90_ 8-fold for imipenem and 8- and ≥16-fold for meropenem, and thus indicating strong potentiation of the in vitro activity (Figure 2B and Figure 3B). Importantly, in the presence of the inhibitor, no carbapenem-resistant strains were detected among the group of CHDL-non-producing *A. baumannii*, with imipenem and meropenem MIC_50/90_ values of ≤0.5/1 and ≤0.5/2 mg/L, i.e. 4- and 8-fold decreases in MIC_90_*,* respectively. The absence of resistance in the presence of LN-1-255 in the latter subset, without acquired CHDLs, could be explained by inhibition of the chromosomal OXA-51, which exhibits weak carbapenemase activity but can contribute to carbapenem resistance to some extent via overexpression mechanisms [8]. Finally, susceptibility to carbapenems was not greatly modified by the combination with the inhibitor in the 59 non-*A. baumannii* isolates as, in all cases, these strains were already fully susceptible to these antibiotics. Of note, the MICs of LN-1-255 alone (MIC > 512 mg/L) indicated that this compound did not exert antimicrobial activity against any of the *Acinetobacter* spp. strains evaluated.

Studies showing the efficacy of inhibitors in recovering the susceptibility to carbapenems in a collection of CHDL-producing carbapenem-resistant *A. baumannii* are scarce. In a recent similar approach to restoring meropenem efficacy against carbapenem-resistant *Acinetobacter* spp., Nelson et al. studied the activity of the combination of the inhibitor QPX7728 (Qpex Biopharma, San Diego, CA, USA) and meropenem against a collection of carbapenem-resistant *A. baumannii* isolates. In a subset of genetically characterized *Acinetobacter* spp. expressing OXA-23 (MIC_50/90_ of 64/64 mg/L for meropenem), the meropenem MIC_50/90_ decreased 32- and 8-fold (i.e., 2/8 mg/L) after the addition of 8 mg/L of QPX7728 [24]. Our findings for the group of acquired CHDL-producing isolates displayed a similar increase in susceptibility, as the addition of LN-1-255 at 8 mg/L decreased the MIC_50/90_ values 8- and ≥16-fold (i.e., 4/4 mg/L), respectively, relative to the values rendered by meropenem alone (Table 2).

Combinations of carbapenems and new β-lactamase inhibitors already in clinical use have also been evaluated in previous studies. Lod et al. and, later, Karlowsky et al. published the results of research aimed at ascertaining the activity of imipenem/relebactam against Gram-negative ESKAPE pathogens isolated from patients in North American and European hospitals [25,26]. The findings showed promising results for both *Enterobacterales* and *Pseudomonas aeruginosa*, but failed to tackle resistance in *A. baumannii*. Similarly, a study of the new meropenem/vaborbactam combination revealed that it was very active against carbapenem-resistant *Enterobacterales* but that the activity was similar to that of meropenem alone against *Acinetobacter* spp. isolates [27]. Thus, the patent inability of these two recent commercially available β-lactamase inhibitors to overcome carbapenem resistance in *Acinetobacter* is underwhelming and, regrettably, exacerbates the urgent clinical need for effective compounds.

Our findings are consistent with those of previous studies testing the inhibition potential of LN-1-255 against *A. baumannii*. Susceptibility assays involving strains harboring the most common CHDLs in this species demonstrated the effectivity of LN-1-255, successfully placing the carbapenems MIC below the resistance clinical breakpoints and, together with inhibition kinetics and docking assays, identifying LN-1-255 as a pan-inhibitor of all *A. baumannii* CHDLs [22]. Moreover, LN-1-255 was able to significantly reduce the bacterial load in the lungs of mice infected with carbapenem-resistant *A. baumannii* strains (carrying either OXA-23 or OXA-24/40) relative to imipenem monotherapy when administered at a dose of 50 mg/kg q3h [23]. Therefore, our results, not only restoring the susceptibility of imipenem but also meropenem in a collection of *Acinetobacter* spp. clinical isolates, add further evidence regarding the suitability of using LN-1-255 in the fight against *Acinetobacter* spp. and specifically carbapenem-resistant *A. baumannii.*

### 2.2. Cefepime/LN-1-255

**Figure 4:**
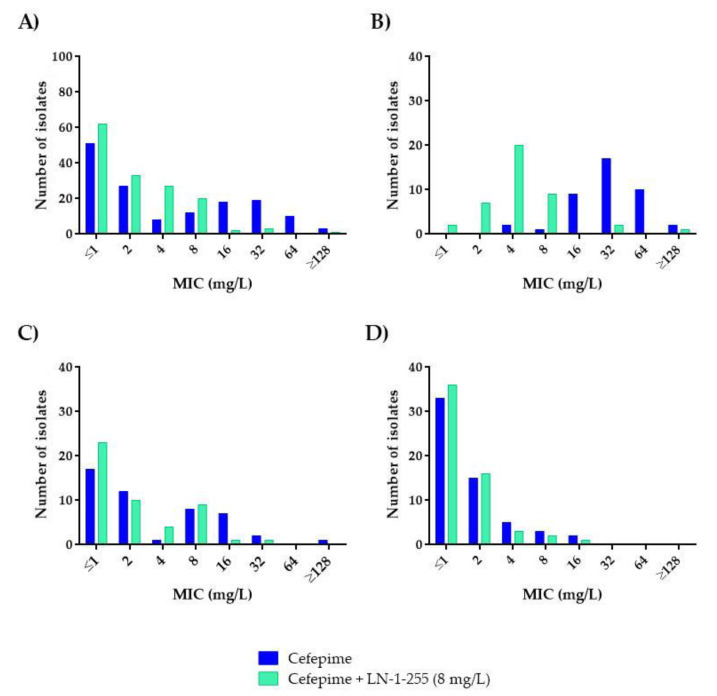
Comparative analysis of cefepime and cefepime/LN-1-255 MICs (mg/L): (**A**) All isolates (*n* = 148), (**B**) CHDL-producing *A. baumannii* (*n* = 41), (**C**) *A. baumannii* producing only OXA-51-like (*n* = 48), and (**D**) non-*A. baumannii* (*n* = 59).

In susceptibility assays, the cefepime MIC_50_ and MIC_90_ values for the 148 *Acinetobacter* spp. isolates were 2 and 32 mg/L, respectively (Table 4). As expected, *A. baumannii* harboring acquired CHDLs yielded the highest MIC_50/90_ values (32/64 mg/L) and the highest rate of resistance (70.7%) to cefepime, followed by the group of *A. baumannii* isolates without CHDLs (2/16 mg/L, 6.3% of resistance) and the non-*A. baumannii* group (≤1/4 mg/L), which did not include any cefepime-resistant isolates (Figure 4). The activity of the cefepime/LN-1-255 combination against the CHDLs-producing *A. baumannii* subset showed the greatest leap, with an 8-fold decrease in MIC_50/90_ (4/8 mg/L) and a consi-derable reduction in resistance rates (from 70.3% to 7.3% in the presence of the inhibitor; Figure 4B). These parameters also decreased in the other two groups of isolates, although less dramatically (MIC_50/90_ of 2/8 mg/L for *A. baumannii* without acquired CHDLs and ≤1/2 mg/L for non-*A. baumannii* isolates).

Hyperproduction of chromosomal OXA-51-like, activation of efflux pumps, and changes in outer membrane porins hinder the efficacy of cefepime against *Acinetobacter*. In addition, the potential acquisition of other CHDLs, extended-spectrum β-lactamases (ESBLs), or metallo-β-lactamases (MBLs) [9,10] a priori rule out cefepime as a treatment option for these pathogens. Interestingly, the MIC of cefepime/LN-1-255 was ≤8 mg/L (the susceptibility breakpoint according to CLSI guidelines) for 38 out of 41 CHDL-producing *A. baumannii* and for 46 out of 48 *A. baumannii* isolates without acquired CHDLs (Figure 4). These findings thus suggest that inhibition of chromosomal OXA-51-like, acquired OXA-type carbapenemases, and, potentially, ESBLs [28] by LN-1-255 may lead to reappraisal of the use of cefepime for *A. baumannii*. Whole-genome sequencing studies will be conducted with the strains of this collection to determine the specific antimicrobial resistance mechanisms carried by isolates.

### 2.3. Sulbactam/LN-1-255

Differences in sulbactam MICs were also observed in the various *Acinetobacter* groups, as found during testing of other antibiotics. As *Acinetobacter* spp. are intrinsically resistant to ampicillin, mainly due to the chromosomal cephalosporinase, the CLSI breakpoints for the ampicillin/sulbactam combination are based on the bactericidal activity of sulbactam against this pathogen. For the whole set of isolates, the sulbactam MIC_50/90_ value was 0.5/8 mg/L. The highest MIC_50/90_ for the *A. baumannii* isolates was observed within the CHDL-producing subset (8/16 mg/L) relative to those lacking CHDLs (0.5/2 mg/L). However, the resistance rates for sulbactam were the lowest among the β-lactam antibiotics tested. Non-*A. baumannii* representatives rendered MIC_50_ and MIC_90_ values of 0.5 and 1 mg/L, respectively (Table 5). When LN-1-255 was combined with sulbactam, the greatest impact on the MIC_50/90_ was again observed in the group of CHDL-producing *A. baumannii* (1/4 mg/L), with 8- and 4-fold decreases in the sulbactam MIC_50_ and MIC_90_ values, respectively. Thus, the rate of susceptibility to sulbactam increased from 36.6% to 97.6% when tested in the presence of the inhibitor (MIC susceptibility breakpoint ≤4 mg/L). No apparent improvement over the use of LN-1-255 was detected among the other *Acinetobacter* spp. included in this study, for which low MICs of sulbactam (alone) were obtained, in all cases, below the clinical susceptibility breakpoints (Table 5).

**Figure 5:**
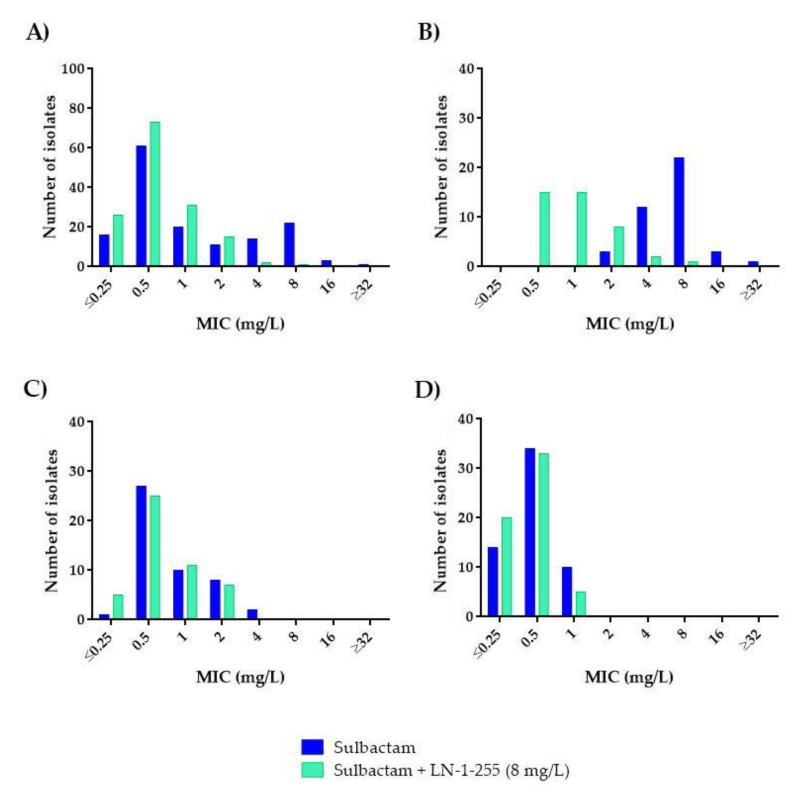
Comparative analysis of sulbactam and sulbactam/LN-1-255 MICs (mg/L): (**A**) All isolates (*n* = 148), (**B**) CHDL-producing *A. baumannii* (*n* = 41), (**C**) *A. baumannii* producing only OXA-51-like (*n* = 48), and (**D**) non-*A. baumannii* (*n* = 59).

As sulbactam remains of potential use, various attempts to assess its therapeutic value have been carried out: in non-life-threatening *A. baumannii* [29] and *A. calcoaceticus* infections [30], in *A. baumannii* borne meningitis [31], and in pharmacodynamic in vitro modeling using human-simulated exposure [32]. In the present study, we observed that in the presence of LN-1-255, the sulbactam MIC values for *Acinetobacter* spp. can probably be reduced to values that would yield therapeutic success in vivo, which would be particularly valuable against carbapenem-resistant isolates (Figure 5).

Studies of any new inhibitors that effectively decrease sulbactam resistance in strains of *A. baumannii* are, again, very scarce. Probably the most remarkable example is the new 1,6-diazabicyclo[3.2.1]octane β-lactamase inhibitor durlobactam (formerly ETX2514, Entasis Therapeutics, Waltham, MA, USA) [33]. The sulbactam/durlobactam combination was tested to exploit the properties of sulbactam, thus bypassing resistance mediated by β-lactamases through the addition of durlobactam. In a study using isolates of carbapenem-resistant *A. baumannii* with different genetic backgrounds, addition of durlobactam at a concentration of 4 mg/L lowered the sulbactam MIC_50_ and MIC_90_ values 16- and 32-fold, respectively [34], placing them below the susceptibility breakpoint (≤4 mg/L). Similar approaches were used to assess the susceptibility of *A. baumannii* to sulbactam/durlobactam in mainland China, with similar results [35].

**Figure 6:**
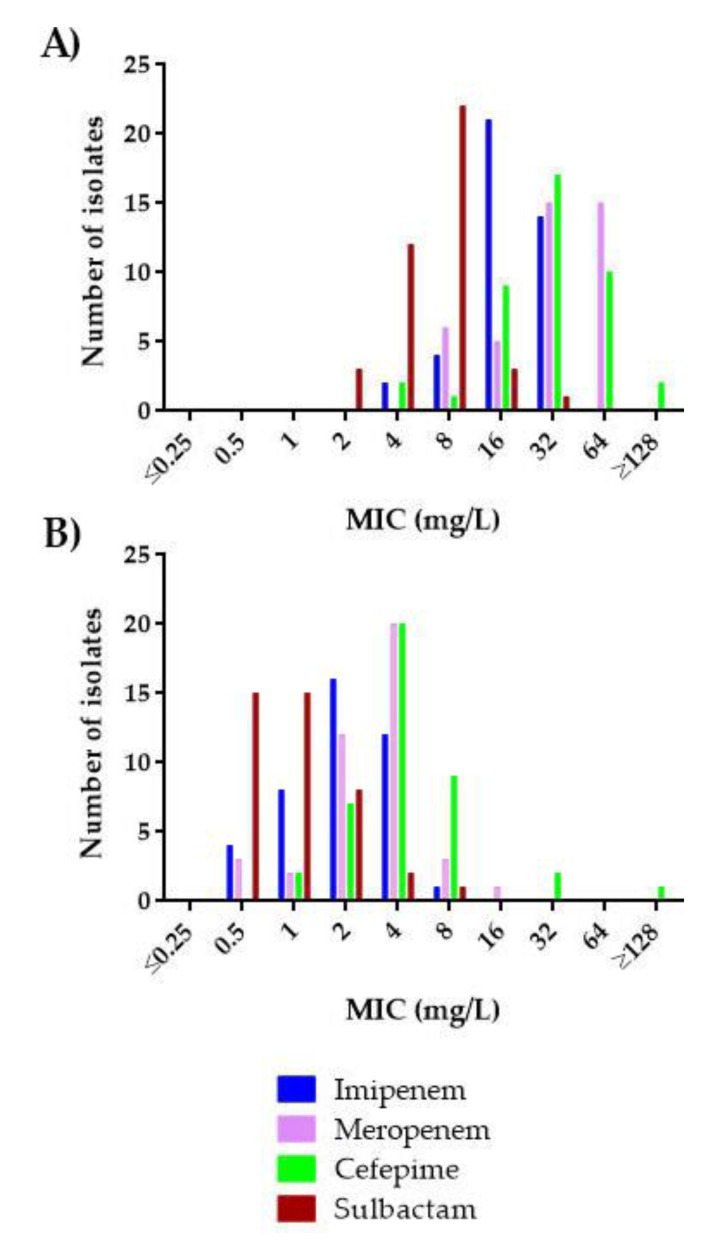
Comparative analysis of the activity of imipenem, meropenem, cefepime, and sulbactam alone (**A**) or in combination with LN-1-255 (**B**) against the set of CHDL-producing *A. baumannii* isolates.

Our findings highlight the strong in vitro activity of the inhibitor LN-1-255 in combination with different β-lactams against the difficult-to-treat carbapenem-resistant CHDL-producing *A. baumannii* isolates (Figure 6). When tested alone, the β-lactam antibiotics (imipenem, meropenem, cefepime, and sulbactam) were weakly active, but when combined with the inhibitor, the MICs decreased greatly. The best results were observed for carbapenems; the susceptible and intermediate rates for imipenem alone increased from 4.9% to 100% in the presence of LN-1-255, and for meropenem, it increased from 0% to 90.2%. However, LN-1-255 also greatly improved the activity of sulbactam, whose susceptibility rates increased from 36.6% to 97.6% when in combination with the inhibitor, and cefepime, increasing the susceptibility from 7.3% to 92.7%.

## 3. Materials and Methods

### 3.1. Bacterial Isolates

Public hospitals in Spain were invited to participate in a nationwide survey of *Acinetobacter* spp. isolates, either from infected patients or as part of colonization studies, for prospective recovery during a 6-month period in 2020. Finally, 14 participated in the survey. Bacterial strains were frozen in Luria-Bertani (LB) broth with 15% glycerol and were maintained at –80 °C until analysis. The clinical microbiological laboratory in the Complexo Hospitalario Universitario A Coruña (A Coruña, Spain), a third-level academic hospital, acted as the reference laboratory.

### 3.2. Antimicrobial Susceptibility Testing

Antimicrobial susceptibility testing was performed by broth microdilution in 96-well microdilution plates, with Mueller-Hinton II broth (Becton, Dickinson and Company, Sparks, MD, USA) and according to the CLSI reference guidelines. Minimum inhibitory concentrations (MICs) were determined for imipenem, meropenem, cefepime, and sulbactam (Sigma, St. Louis, MO, USA) alone or in combination with the inhibitor LN-1-255 at a fixed concentration of 8 mg/L. LN-1-255 was synthesized at the Center for Research in Biological Chemistry and Molecular Materials (CIQUS, University of Santiago of Compostela, Santiago de Compostela, Spain), as previously reported [36]. The 2020 CLSI clinical breakpoints and guidelines (CLSI M100 ED30:2020) were used for interpretation [37]. We adopted a breakpoint of ≤4 mg/L for susceptibility (S), 8 mg/L for intermediate susceptibility (I), and ≥16 mg/L for resistance (R) to sulbactam alone (no breakpoints available), based on that of the ampicillin/sulbactam combination, reported by the CLSI (≤8/4 mg/L, S; 16/8 mg/L, I; and ≥32/16 mg/L, R).

### 3.3. Multiplex PCR Assay 

Multiplex PCR was used to identify the oxacillinase-encoding genes expressed by the entire set of isolates, as previously described [38]. The primers used to identify *bla*_oxa-23-like_, *bla*_OXA-24/40-like_, *bla*_OXA-51-like_, *bla*_OXA-58-like,_ *bla*_OXA-143-like_, and *bla*_OXA-235-like_ are listed in Table 6. 

## 4. Conclusions

In this study, we evaluated for the first time the efficacy of the penicillin sulfone LN-1-255 in combination with several β-lactam antibiotics against a collection of clinical isolates of *Acinetobacter* spp. isolated in a multicenter study. Our findings highlight the ability of the β-lactamase inhibitor LN-1-255 to restore the efficacy of imipenem and meropenem as first-line antibiotics in the fight against *A. baumannii* infections and identify this inhibitor as one of the very few in development that is able to block CHDLs produced by this bacterium. Use of LN-1-255 also brings compounds such as cefepime and (notably) sulbactam back into play in the effort to diminish the selective pressure derived from overuse of carbapenems.

## Figures and Tables

**Table 1:** Susceptibility of *Acinetobacter* spp. isolates to imipenem and imipenem/LN-1-255.

Isolates	Imipenem						Imipenem/LN-1-255					
	MIC (mg/L)			CLSI Category			MIC (mg/L)			CLSI Category		
	MIC_50_	MIC_90_	Range	%S	%I	%R	MIC_50_	MIC_90_	Range	%S	%I	%R
All isolates (*n* = 148)	≤0.5	16	≤0.5 to 32	64.9	3.8	28.4	≤0.5	2	≤0.5 to 4	91.2	8.1	0.0
CHDL-producing *A. baumannii* (*n* = 41)	16	32	4 to 32	0.0	4.9	95.1	2	4	≤0.5 to 4	68.3	31.7	0.0
Non-CHDL-producing *A. baumannii* (*n* = 48)	≤0.5	4	≤0.5 to 16	77.1	16.7	6.3	≤0.5	1	≤0.5 to 2	100	0.0	0.0
Non-*A. baumannii* (*n* = 59)	≤0.5	≤0.5	≤0.5	100	0.0	0.0	≤0.5	≤0.5	≤0.5	100	0.0	0.0

S, susceptible; I, intermediate; R, resistant.

**Table 2:** Susceptibility of *Acinetobacter* spp. isolates to meropenem and meropenem/LN-1-255.

Isolates	Meropenem						Meropenem/LN-1-255					
	MIC (mg/L)			CLSI Category			MIC (mg/L)			CLSI Category		
	MIC_50_	MIC_90_	Range	%S	%I	%R	MIC_50_	MIC_90_	Range	%S	%I	%R
All isolates (*n* = 148)	≤0.5	32	≤0.5 to ≥64	64.9	0.0	35.1	≤0.5	4	≤0.5 to 16	81.1	16.2	2.7
CHDL-producing *A. baumannii* (*n* = 41)	32	≥64	8 to ≥64	0.0	0.0	100	4	4	≤0.5 to 16	41.5	48.7	9.8
Non-CHDL-producing *A. baumannii* (*n* = 48)	≤0.5	16	≤0.5 to 16	77.1	0.0	22.9	≤0.5	2	≤0.5 to 4	91.7	8.3	0.0
Non-*A. baumannii* (*n* = 59)	≤0.5	≤0.5	≤0.5	100	0.0	0.0	≤0.5	≤0.5	≤0.5	100	0.0	0.0

S, susceptible; I, intermediate; R, resistant.

**Table 3:** Identification of CHDLs between 41 CHDLs-producing *A. baumannii.*

CHDLs	Number of *A. baumannii* Isolates Producing CHDLs (%)
OXA-23-like	34 (82.92%)
OXA-24-like	0
OXA-58-like	7 (17.08%)
OXA-148-like	0
OXA-235-like	0

**Table 4:** Susceptibility of *Acinetobacter* spp. isolates to cefepime and cefepime/LN-1-255.

Isolates	Cefepime						Cefepime/LN-1-255					
	MIC (mg/L)			CLSI Category			MIC (mg/L)			CLSI Category		
	MIC_50_	MIC_90_	Range	%S	%I	% R	MIC_50_	MIC_90_	Range	%S	%I	%R
All isolates (*n* = 148)	2	32	≤1 to ≥128	66.2	12.2	21.6	2	8	≤1 to ≥128	95.9	1.4	2.7
CHDL-producing *A. baumannii* (*n* = 41)	32	64	4 to ≥128	7.3	22.0	70.7	4	8	≤1 to ≥128	92.7	0.0	7.3
Non-CHDL-producing *A. baumannii* (*n* = 48)	2	16	≤1 to ≥128	79.2	14.6	6.3	2	8	≤1 to 32	95.8	2.1	2.1
Non-*A. baumannii* (*n* = 59)	≤1	4	≤1 to 16	96.6	3.4	0.0	≤ 1	2	≤1 to 16	98.3	1.7	0.0

S, susceptible; I, intermediate; R, resistant.

**Table 5:** Susceptibility of *Acinetobacter* spp. isolates to sulbactam and sulbactam/LN-1-255.

Isolates	Sulbactam						Sulbactam/LN-1-255					
	MIC (mg/L)			CLSI Category			MIC (mg/L)			CLSI Category		
	MIC_50_	MIC_90_	Range	%S	%I	%R	MIC_50_	MIC_90_	Range	%S	%I	%R
All isolates (*n* = 148)	0.5	8	≤0.25 to ≥32	82.4	14.9	2.7	0.5	2	≤0.25 to 8	99.3	0.7	0.0
CHDL-producing *A.* *baumannii* (*n* = 41)	8	16	2 to ≥32	36.6	53.7	9.8	1	4	0.5 to 8	97.6	2.4	0.0
Non-CHDL-producing *A. baumannii* (*n* = 48)	0.5	2	≤0.25 to 4	100	0.0	0.0	0.5	2	≤0.25 to 2	100	0.0	0.0
Non-*A. baumannii* (*n* = 59)	0.5	1	≤0.25 to 1	100	0.0	0.0	0.5	0.5	≤0.25 to 1	100	0.0	0.0

S, susceptible; I, intermediate; R, resistant.

**Table 6:** Primers used in the study.

Primer	Sequence (5′-3′)	Product Size (pb)	Reference
OXA-23likeFw	GATCGGATTGGAGAACCAGA	501	[38]
OXA-23likeRv	ATTTCTGACCGCATTTCCAT		
OXA-24/40likeFw	GGTTAGTTGGCCCCCTTAAA	246	[38]
OXA-24/40likeRv	AGTTGAGCGAAAAGGGGATT		
OXA-51likeFw	TAATGCTTTGATCGGCCTTG	353	[38]
OXA-51likeRv	TGGATTGCACTTCATCTTGG		
OXA-58likeFw	AAGTATTGGGGCTTGTGCTG	599	[38]
OXA-58likeRv	CCCCTCTGCGCTCTACATAC		
OXA-143likeFw	TACAACAACTGAGATTTTCA	390	This study
OXA-143likeRv	GGGGTTACATCCATTCC		
OXA-235likeFw	ATGGGATGGCAAGAAGC	239	This study
OXA-235likeRv	GAGGCAAATTCGACTTCT		

## Data Availability

Not applicable.

## References

[1] Joly-Guillou M.-L. (2005). Clinical impact and pathogenicity of Acinetobacter. Clin. Microbiol. Infect..

[2] Lupo A., Haenni M., Madec J.-Y. (2018). Antimicrobial Resistance in Acinetobacter spp. and Pseudomonas spp. Antimicrob. Resist. Bact. Livest. Companion Anim..

[3] Murray G.L., Peleg A.Y., Doi Y. (2015). Acinetobacter baumannii: Evolution of Antimicrobial Resistance—Treatment Options. Semin. Respir. Crit. Care Med..

[4] Pérez A., Pérez-Llarena F.J., García P., Kerff F., Beceiro A., Galleni M., Bou G. (2014). New mutations in ADC-type β-lactamases from Acinetobacter spp. affect cefoxitin and ceftazidime hydrolysis. J. Antimicrob. Chemother..

[5] Urban C., Go E., Mariano N., Rahal J.J. (1995). Interaction of sulbactam, clavulanic acid and tazobactam with penicillin-binding proteins of imipenem-resistant and -susceptible acinetobacter baumannii. FEMS Microbiol. Lett..

[6] Mendes R.E., Bell J.M., Turnidge J.D., Castanheira M., Jones R.N. (2008). Emergence and widespread dissemination of OXA-23, -24/40 and -58 carbapenemases among Acinetobacter spp. in Asia-Pacific nations: Report from the SENTRY Surveillance Program. J. Antimicrob. Chemother..

[7] Rumbo C., Gato E., López M., De Alegría C.R., Fernández-Cuenca F., Martínez-Martínez L., Vila J., Pachón J., Cisneros J.M., Rodríguez-Baño J. (2013). Contribution of Efflux Pumps, Porins, and β-Lactamases to Multidrug Resistance in Clinical Isolates of Acinetobacter baumannii. Antimicrob. Agents Chemother..

[8] Viehman J.A., Nguyen M.-H., Doi Y. (2014). Treatment Options for Carbapenem-Resistant and Extensively Drug-Resistant Acinetobacter baumannii Infections. Drugs.

[9] Tian G.-B., Adams-Haduch J.M., A Taracila M., Bonomo R.A., Wang H.-N., Doi Y. (2011). Extended-Spectrum AmpC Cephalosporinase in Acinetobacter baumannii: ADC-56 Confers Resistance to Cefepime. Antimicrob. Agents Chemother..

[10] Endimiani A., Perez F., A Bonomo R. (2008). Cefepime: A reappraisal in an era of increasing antimicrobial resistance. Expert Rev. Anti-Infect. Ther..

[11] Yang Y., Xu Q., Li T., Fu Y., Shi Y., Lan P., Zhao D., Chen Q., Zhou Z., Jiang Y. (2018). OXA-23 Is a Prevalent Mechanism Contributing to Sulbactam Resistance in Diverse Acinetobacter baumannii Clinical Strains. Antimicrob. Agents Chemother..

[12] Yang Y., Fu Y., Lan P., Xu Q., Jiang Y., Chen Y., Ruan Z., Ji S., Hua X., Yu Y. (2018). Molecular Epidemiology and Mechanism of Sulbactam Resistance in Acinetobacter baumannii Isolates with Diverse Genetic Backgrounds in China. Antimicrob. Agents Chemother..

[13] Fernández-Cuenca F., Martínez-Martínez L., Conejo M.C., Ayala J.A., Perea E.J., Pascual A. (2003). Relationship between beta-lactamase production, outer membrane protein and penicillin-binding protein profiles on the activity of carbapenems against clinical isolates of Acinetobacter baumannii. J. Antimicrob. Chemother..

[14] Karlowsky J.A., Kazmierczak K.M., Bouchillon S.K., De Jonge B.L.M., Stone G.G., Sahm D.F. (2018). In Vitro Activity of Ceftazidime-Avibactam against Clinical Isolates of Enterobacteriaceae and Pseudomonas aeruginosa Collected in Asia-Pacific Countries: Results from the INFORM Global Surveillance Program, 2012 to 2015. Antimicrob. Agents Chemother..

[15] Bush K., Jacoby G.A., Medeiros A.A. (1995). A functional classification scheme for beta-lactamases and its correlation with molecular structure. Antimicrob. Agents Chemother..

[16] Tooke C.L., Hinchliffe P., Bragginton E.C., Colenso C.K., Hirvonen V.H., Takebayashi Y., Spencer J. (2019). β-Lactamases and β-Lactamase Inhibitors in the 21st Century. J. Mol. Biol..

[17] González-Bello C., Rodríguez D., Pernas M., Rodríguez Á., Colchón E. (2020). β-Lactamase Inhibitors to Restore the Efficacy of Antibiotics against Superbugs. J. Med. Chem..

[18] Shapiro A.B., Gao N., Jahić H., Carter N.M., Chen A., Miller A.A. (2017). Reversibility of Covalent, Broad-Spectrum Serine β-Lactamase Inhibition by the Diazabicyclooctenone ETX2514. ACS Infect. Dis..

[19] Tsivkovski R., Totrov M., Lomovskaya O. (2020). Biochemical Characterization of QPX7728, a New Ultrabroad-Spectrum Beta-Lactamase Inhibitor of Serine and Metallo-Beta-Lactamases. Antimicrob. Agents Chemother..

[20] Drawz S.M., Bethel C.R., Doppalapudi V.R., Sheri A., Pagadala S.R.R., Hujer A.M., Skalweit M.J., Anderson V.E., Chen S.G., Buynak J.D. (2010). Penicillin Sulfone Inhibitors of Class D β-Lactamases. Antimicrob. Agents Chemother..

[21] Rodríguez D., Maneiro M., Vázquez-Ucha J.C., Beceiro A., González-Bello C. (2020). 6-Arylmethylidene Penicillin-Based Sulfone Inhibitors for Repurposing Antibiotic Efficiency in Priority Pathogens. J. Med. Chem..

[22] Vázquez-Ucha J.C., Maneiro M., Martínez-Guitián M., Buynak J., Bethel C.R., Bonomo R.A., Bou G., Poza M., González-Bello C., Beceiro A. (2017). Activity of the β-Lactamase Inhibitor LN-1-255 against Carbapenem-Hydrolyzing Class D β-Lactamases from Acinetobacter baumannii. Antimicrob. Agents Chemother..

[23] Vázquez-Ucha J.C., Martínez-Guitián M., Maneiro M., Conde-Pérez K., Álvarez-Fraga L., Torrens G., Oliver A., Buynak J.D., Bonomo R.A., Bou G. (2019). Therapeutic Efficacy of LN-1-255 in Combination with Imipenem in Severe Infection Caused by Carbapenem-Resistant Acinetobacter baumannii. Antimicrob. Agents Chemother..

[24] Nelson K., Rubio-Aparicio D., Tsivkovski R., Sun D., Totrov M., Dudley M., Lomovskaya O. (2020). In Vitro Activity of the Ultra-Broad-Spectrum Beta-lactamase Inhibitor QPX7728 in Combination with Meropenem against Clinical Isolates of Carbapenem-Resistant Acinetobacter baumannii. Antimicrob. Agents Chemother..

[25] Lob S.H., Hackel M.A., Kazmierczak K.M., Young K., Motyl M.R., Karlowsky J.A., Sahm D.F. (2017). In Vitro Activity of Imipenem-Relebactam against Gram-Negative ESKAPE Pathogens Isolated by Clinical Laboratories in the United States in 2015 (Results from the SMART Global Surveillance Program). Antimicrob. Agents Chemother..

[26] A Karlowsky J., Lob S.H., Kazmierczak K.M., Hawser S.P., Magnet S., Young K., Motyl M.R., Sahm D.F. (2018). In vitro activity of imipenem/relebactam against Gram-negative ESKAPE pathogens isolated in 17 European countries: 2015 SMART surveillance programme. J. Antimicrob. Chemother..

[27] Castanheira M., Huband M.D., Mendes R.E., Flamm R.K. (2017). Meropenem-Vaborbactam Tested against Contemporary Gram-Negative Isolates Collected Worldwide during 2014, Including Carbapenem-Resistant, KPC-Producing, Multidrug-Resistant, and Extensively Drug-Resistant Enterobacteriaceae. Antimicrob. Agents Chemother..

[28] Pattanaik P., Bethel C.R., Hujer A.M., Hujer K.M., Distler A.M., Taracila M., Anderson V.E., Fritsche T.R., Jones R.N., Pagadala S.R.R. (2009). Strategic Design of an Effective β-Lactamase Inhibitor. J. Biol. Chem..

[29] Hancock R.E.W. (1998). Resistance Mechanisms inPseudomonas aeruginosaand Other Nonfermentative Gram-Negative Bacteria. Clin. Infect. Dis..

[30] Urban C., Go E., Mariano N., Berger B.J., Avraham I., Rubin D., Rahal J.J. (1993). Effect of Sulbactam on Infections Caused by Imipenem-ResistantAcinetobacter calcoaceticusBiotypeanitratus. J. Infect. Dis..

[31] Jimenez-Mejias M.E., Pachon J., Becerril B., Palomino-Nicas J., Rodriguez-Cobacho A., Revuelta M. (1997). Treatment of Multidrug-Resistant Acinetobacter baumannii Meningitis with Ampicillin/Sulbactam. Clin. Infect. Dis..

[32] Housman S.T., Hagihara M., Nicolau D.P., Kuti J.L. (2013). In vitro pharmacodynamics of human-simulated exposures of ampicillin/sulbactam, doripenem and tigecycline alone and in combination against multidrug-resistant Acinetobacter baumannii. J. Antimicrob. Chemother..

[33] Durand-Réville T.F., Guler S., Comita-Prevoir J., Chen B., Bifulco N., Huynh H., Lahiri S., Shapiro A.B., McLeod S.M., Carter N.M. (2017). ETX2514 is a broad-spectrum β-lactamase inhibitor for the treatment of drug-resistant Gram-negative bacteria including Acinetobacter baumannii. Nat. Microbiol..

[34] Seifert H., Müller C., Stefanik D., Higgins P.G., Miller A., Kresken M. (2020). In vitro activity of sulbactam/durlobactam against global isolates of carbapenem-resistant Acinetobacter baumannii. J. Antimicrob. Chemother..

[35] Yang Q., Xu Y., Jia P., Zhu Y., Zhang J., Zhang G., Deng J., Hackel M., A Bradford P., Reinhart H. (2020). In vitro activity of sulbactam/durlobactam against clinical isolates of Acinetobacter baumannii collected in China. J. Antimicrob. Chemother..

[36] Buynak J.D., Rao A., Doppalapudi V.R., Adam G., Petersen P.J., Nidamarthy S.D. (1999). The synthesis and evaluation of 6-alkylidene-2’β-substituted penam sulfones as β-lactamase inhibitors. Bioorganic Med. Chem. Lett..

[37] CLSI (2020). Performance Standards for Antimicrobial Susceptibility Testing.

[38] Woodford N., Ellington M.J., Coelho J.M., Turton J.F., Ward M.E., Brown S., Amyes S.G., Livermore D.M. (2006). Multiplex PCR for genes encoding prevalent OXA carbapenemases in Acinetobacter spp. Int. J. Antimicrob. Agents.

